# Altered Effective Connectivity of the Primary Motor Cortex in Stroke: A Resting-State fMRI Study with Granger Causality Analysis

**DOI:** 10.1371/journal.pone.0166210

**Published:** 2016-11-15

**Authors:** Zhiyong Zhao, Xiangmin Wang, Mingxia Fan, Dazhi Yin, Limin Sun, Jie Jia, Chaozheng Tang, Xiaohui Zheng, Yuwei Jiang, Jie Wu, Jiayu Gong

**Affiliations:** 1 Shanghai Key Laboratory of Magnetic Resonance, East China Normal University, Shanghai, 200062, China; 2 Institute of Neuroscience, State Key Laboratory of Neuroscience, Key Laboratory of Primate Neurobiology, CAS Center for Excellence in Brain Science and Intelligence Technology, Shanghai Institute for Biological Sciences, Chinese Academy of Sciences, Shanghai, 200031, China; 3 Department of Rehabilitation, Huashan Hospital, Fudan University, Shanghai, 200040, China; University of Texas at Austin, UNITED STATES

## Abstract

The primary motor cortex (M1) is often abnormally recruited in stroke patients with motor disabilities. However, little is known about the alterations in the causal connectivity of M1 following stroke. The purpose of the present study was to investigate whether the effective connectivity of the ipsilesional M1 is disturbed in stroke patients who show different outcomes in hand motor function. 23 patients with left-hemisphere subcortical stroke were selected and divided into two subgroups: partially paralyzed hands (PPH) and completely paralyzed hands (CPH). Further, 24 matched healthy controls (HCs) were recruited. A voxel-wise Granger causality analysis (GCA) on the resting-state fMRI data between the ipsilesional M1 and the whole brain was performed to explore differences between the three groups. Our results showed that the influence from the frontoparietal cortices to ipsilesional M1 was diminished in both stroke subgroups and the influence from ipsilesional M1 to the sensorimotor cortices decreased greater in the CPH group than in the PPH group. Moreover, compared with the PPH group, the decreased influence from ipsilesional M1 to the contralesional cerebellum and from the contralesional superior parietal lobe to ipsilesional M1 were observed in the CPH group, and their GCA values were positively correlated with the FMA scores; Conversely, the increased influence from ipsilesional M1 to the ipsilesional middle frontal gyrus and middle temporal gyrus were observed, whose GCA values were negatively correlated with the FMA scores. This study suggests that the abnormalities of casual flow in the ipsilesional M1 are related to the severity of stroke-hand dysfunction, providing valuable information to understand the deficits in resting-state effective connectivity of motor execution and the frontoparietal motor control network during brain plasticity following stroke.

## Introduction

Stroke is a leading cause of adult disability and has a stable and high death rate [1]. Motor disability is one of the most common deficits observed in stroke patients [2], and impairments in hand function have a particularly serious negative impact on daily activities and the quality of life.

Functional magnetic resonance imaging (fMRI) has contributed to our understanding of the pathophysiology of stroke by examining the functional reorganization of the motor system [3–9]. Compared with task-based fMRI, resting-state fMRI is a more attractive approach because it can be performed without the patient doing any task or external input. This type of analysis is more suitable for studying the reorganization of the cerebral cortex in stroke patients with severe hand function disabilities. Hand movement is most closely related to the primary motor cortex (M1), which is located in the precentral gyrus [10]. In recent years, resting-state fMRI studies of cerebral cortical reorganization in stroke patients have primarily focused on M1 [4, 7, 9]. Park et al [4] conducted a longitudinal study on stroke patients using resting-state functional connectivity (FC) with the ipsilesional M1 as a seed region. They found an increase in the FC of the ipsilesional M1 with the ipsilesional frontal and parietal cortices, bilateral thalamus, and cerebellum, and a decreased FC in the contralesional M1 and the occipital cortex. Our previous study performed an FC analysis on both the ipsilesional and contralesional M1, and observed abnormal connectivity with many motor brain areas in stroke patients [9]. A network-level analysis of functional reorganization following stroke has also reported an increase in the regional centralities of the ipsilesional M1 and contralesional cerebellum [7]. However, the methods used in the aforementioned studies cannot reveal the direction of the FC between the brain regions involved.

The importance of effective connectivity, which is defined as the influence of one neuronal system on another, has become increasingly recognized. Two common approaches for studying effective connectivity have been used in stroke patients with motor disabilities. For example, Inman et al [11] utilized structural equation modeling (SEM) to investigate the effective connectivity between brain regions that are involved in motor control and motor execution, and they found diminished connectivity from the superior parietal cortex to M1 and the supplementary motor cortex. The causal interaction between cortical motor areas during hand movements has also been assessed using dynamic causal modeling (DCM) in patients with subcortical stroke [12–16]. However, these models require assumptions about the existence and direction of influence between any two regions; therefore, any misspecification of the models may result in erroneous conclusions [17]. Granger causality analysis (GCA) is another effective connectivity method that originates from the field of economics, and can be used to measure the causal influence and flow of information for fMRI time-series. In addition, GCA provides information about the dynamics and directionality of fMRI BOLD signal in cortical circuits [18, 19] (for more details of the GCA rationale see the Materials and Methods). GCA effectively compensates for the shortcomings of the two common approaches above, as there is no need for any prior knowledge [17]. Also, GCA has been employed to detect the abnormal effective connectivity in widespread diseases [18, 20–23]. Using a multivariate GCA, Hamilton et al [20] demonstrated the directionality of influence within abnormal resting-state networks in major depressive disorder. Ji et al [21] revealed a disruption in causal connectivity by the GCA in cases of mesial temporal lobe epilepsy. Liao et al [22] used GCA on resting-state fMRI data to investigate a network of effective connectivity associated with the amygdala in social anxiety disorder; however, this method is rarely used to study stroke patients with motor disabilities.

Taking into account the important role of M1 in hand motor recovery following stroke [4, 7, 9], we explored the resting-state effective connectivity between the ipsilesional M1 and the whole brain using a voxel-wise GCA in stroke patients. According to the different outcomes in hand function, we divided the patients into two subgroups: one with partially paralyzed hands (PPH) and the other with completely paralyzed hands (CPH) [9]. Thus, we explored the relationship between hand motion disability and effective connectivity. We hypothesized that the effective connectivity of the ipsilesional M1 would be affected in stroke patients. And, compared with healthy controls (HCs), the PPH and CPH groups would exhibit different reorganization patterns of the effective connectivity of the ipsilesional M1. Furthermore, we tested whether the effective connectivity of the abnormal brain regions correlates with the outcomes in hand function.

## Materials and Methods

### Participants

Twenty-three subcortical stroke patients (all right handed; aged 48–76 years) with pure motor deficits were recruited from Huashan Hospital, which is affiliated with Fudan University. All patients underwent testing using the Mini-Mental State Examination (MMSE) and Fugl-Meyer Assessment (FMA). The inclusion criteria were as follows: (1) first-ever subcortical stroke in the left basal ganglia; (2) at least 6 months after stroke, all in the sequela phase after the recovery period; (3) sufficient cognitive abilities (MMSE > 24); (4) right hand motor deficit. The stroke patients were divided into two subgroups: the PPH group (12 patients, 11 males, age (Mean ±SD): 62 ±7.6 years) and the CPH group (11 patients, 8 males, age (Mean ± SD): 62 ±6.5 years). This classification was based on our assessment of their paralyzed hand function involving five practical actions of the hand in daily life (S1 Table). Individuals who could not complete any of the actions were placed in the CPH group, while the others, who could complete at least one of the five actions, were placed in the PPH group [9]. The assessments were performed by two experienced physicians in the Department of Rehabilitation Medicine, Huashan Hospital. The clinical characteristics and demographic data are summarized in Table 1. Twenty-four age- and sex-matched healthy controls (14 males, age (Mean ± SD): 62 ±9.8 years) were recruited from local communities. All participants with (1) a contraindication to MRI; (2) severe quadriplegia; (3) a history of neurological and psychiatric disorders; or (4) a history of hand dysfunction were excluded.

**Table 1:** Clinical and demographic data of 23 subcortical stroke patients.

Subject	Sex	Age(yr)	Location of Lesion	course of disease (months)	MMSE	FMA Scores (hand+wrist)
**PPH group**						
1	M	56	L,IC,BG,Th	14	30	23
2	M	60	L,IC,Th^a^	53	30	12
3	F	48	L,IC,BG	23	29	6
4	M	76	L,IC	21	27	22
5	M	60	L,IC,BG	36	30	6
6	M	71	L,IC,Th^a^	22	27	11
7	M	65	L,IC,BG,Th	6	28	14
8	M	62	L,IC,Th^a^	6	27	22
9	M	60	L,BG,Th^a^	11	30	23
10	M	65	L,IC,Th	12	29	23
11	M	53	L,IC,BG,Th^a^	22	26	15
12	M	65	L,IC,BG^a^	6	29	20
**CPH group**						
1	M	62	L,BG,IC,Th	6	28	4
2	M	56	L,IC	7	27	6
3	M	56	L,BG,IC,Th^a^	21	27	1
4	M	57	L,IC,Th	19	28	0
5	F	75	L,IC,CR	24	29	4
6	M	63	L,BG,IC,Th^a^	16	28	1
7	F	65	L,IC,Th	17	30	1
8	M	68	L,IC,Th^a^	47	29	1
9	M	61	L,BG,CR	6	30	2
10	F	50	L,BG,IC,Th^a^	13	28	0
11	M	61	L,IC,BG	6	29	4

Abbreviations: M = male; F = female; L = left; R = right; BG = basal ganglia; IC = internal capsule; Th = thalamus; CR = coronal radiata; FMA = Fugl-Meyer Assessment; MMSE = Mini-Mental State Examination.

^a^The characteristics of the lesion are hemorrhagic; others are ischemic.

This study was approved by the Institutional Ethics Committee of East China Normal University, and all participants signed informed consent forms.

### Data Acquisition

All data were acquired using a Siemens Trio 3.0 Tesla MRI scanner (Siemens, Erlangen, Germany) at the Shanghai Key Laboratory of Magnetic Resonance, East China Normal University. We used foam pads to fix the head of each participant to reduce both head movements and scanner noise. Resting-state functional images were acquired using an echo-planar imaging (EPI) sequence (30 transverse slices, slice thickness/gap = 4 mm/0.8 mm, matrix = 64×64, repetition time = 2000 ms, echo time = 30 ms, flip angle = 90°, field of view = 220 mm×220 mm). Structural images of T1-weighted anatomical images in a sagittal orientation were obtained using magnetization prepared by rapid gradient echo sequence (MPRAGE) (192 slices covered the whole brain, slice thickness/gap = 1 mm/0.5 mm, repetition time = 1900 ms, echo time = 3.42 ms, field of view = 240 mm×240 mm, matrix = 256×256). To identify the location of the lesion, T2-weighted images were collected using a turbo-spin-echo sequence (30 axial slices, thickness = 5 mm, no gap, repetition time = 6000 ms, echo time = 93 ms, field of view = 220 mm×220 mm, matrix = 320×320). During the resting-state functional MRI collection, the participants were instructed to close their eyes but to remain awake while trying not to think about anything specific. We collected a total of 240 image volumes.

### Lesion Mapping

**Fig 1:**
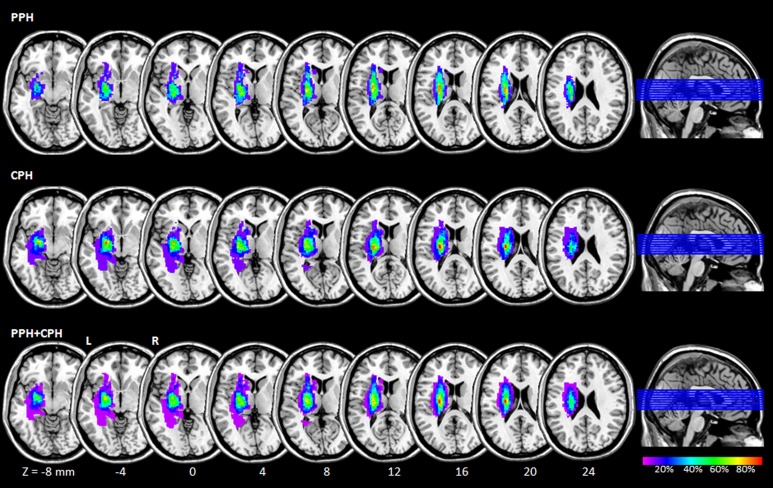
Lesion overlap map across the 23 stroke patients. Color coding indicates the percentage of lesion overlap. Z-axis from Z = -8 to Z = 24 in MNI coordinates, with an incremental interval of 4. R: right; L: left; MNI: Montreal Neurological Institute; PPH: partially paralyzed hands; CPH: completely paralyzed hands.

We constructed a lesion overlap image for stroke patients. The lesion location of each patient which was determined by T2-weighted imaging was shown in S1 Fig. Then we manually outlined the profiles on individual T2-weighted MR images slice by slice using the software MRIcron (http://www.mccauslandcenter.sc.edu/mricro/mricron/), thereby creating a lesion mask for each patient. After the spatial normalization process, the union of all individual lesion masks was used to construct a group lesion mask for the patients (Fig 1).

### Data Analysis

Preprocessing of the fMRI data was performed using the DPARSF (http://www.restfmri.net) and SPM8 (http://www.fil.ion.ucl.ac.uk/spm) toolkits. The first 10 functional volumes were discarded to ensure steady-state longitudinal magnetization. The remaining 230 volumes were slice-time corrected relative to the middle axial slice to account for the temporal difference in acquisition among the different slices, and volumes were then registered to correct for head motion during the scan. No translation or rotation parameters in any given data set exceeded 2 mm or 2°. The functional images were then spatially normalized to standard stereotaxic coordinates from the standard Montreal Neurological Institute (MNI), resampled into a voxel size of 3 × 3 × 3 mm^3^, and then smoothed by convolution with an isotropic Gaussian kernel at a full width at a half maximum (FWHM) of 6 mm to decrease spatial noise. Finally, we removed linear trends from the time courses and used temporal band-pass filtering (0.01–0.08 Hz) to remove the effects of low-frequency drift and high-frequency noise, such as respiratory and heart rhythms.

### Definition of the seed region of interest (ROI)

In the present study, we focused on the hand motor dysfunction of stroke patients. We selected the hand representation of the ipsilesional (left) M1 as the seed ROI for resting-state effective connectivity analysis, which was defined as a sphere with a radius of 6 mm and centered at the peak MNI coordinates of -38, -22, 56 [7].

### Effective connectivity analysis

First, nine nuisance covariates (six head motion parameters, the global signal, white matter signal, and cerebrospinal fluid signal) were removed following preprocessing. Then, we used GCA to calculate the effective connectivity between the reference time series of the ROI and the time series of each voxel in the whole brain. The voxel-wise GCA was performed using the REST-GCA in the REST toolbox (http://www.restfmri.net). GCA was originally proposed in the field of economics to evaluate causal relationships between two time series and states that, if the current value of time course Y could be more accurately estimated by the combination of the past value of time courses X and Y than by the past value of Y alone, then X has a Granger causal influence on Y [24]. Granger causality is often used for fMRI data analysis via Vector Auto-Regression as follows:

$$\begin{array}{l} Y_{t}=\sum_{k=1}^{p}A_{k}X_{\left(t-k\right)}+\sum_{k=1}^{p}B_{k}Y_{\left(t-k\right)}+CZ_{t}+E_{t}\\ X_{t}=\sum_{k=1}^{p}A_{k}^{'}Y_{\left(t-k\right)}+\sum_{k=1}^{p}B_{k}^{'}X_{\left(t-k\right)}+C^{'}Z_{t}+E^{'}{}_{t} \end{array}$$

where *X*_*t*_ and *Y*_*t*_ represent two time series, *A*_*k*_ and *A´*_*k*_ are signed-path coefficients, *B*_*k*_ and *B´*_*k*_ are auto-regression coefficients, *E*_*t*_ and *E´*_*t*_ are residual, and *Z*_*t*_ represents covariates (e.g., head motion, global trend, and time series from certain brain areas). The time series *X*_*t*_ significantly causes the time series *Y*_*t*_ if the signed-path coefficient *A*_*k*_ is significantly larger. Likewise, *Y*_*t*_ can be defined as a significant Granger cause to *X*_*t*_ if the signed-path coefficient *A´*_*k*_ is significantly larger [24]. In the current study, the time series of the ipsilesional M1 was defined as the time series X, and the time course of each voxel in the whole brain was defined as Y. We carried out a bivariate coefficient GCA to investigate the Granger causal influence between the seed ROI and each voxel of the whole brain. Finally, the GCA maps for all subjects were converted to z-values using Fisher’s r-to-z transformation to improve the normality.

Within-group analysis for effective connectivity of the ipsilesional M1 was conducted using a one-sample t-test which compared the z-value of individual voxel with a normal distribution with mean of zero and an unknown variance. Furthermore, two-sample t-tests were conducted to detect the altered effective connectivity between each pair of the three groups (AlphaSim corrected significance level of P < 0.05 was obtained by clusters with a minimum volume of 1080 mm^3^ and individual voxel height threshold of P < 0.01).

### Correlation analysis between effective connectivity and clinical scores

To explore whether the abnormal effective connectivity of the ipsilesional M1 in stroke patients was related to hand function, we performed a Pearson correlation analysis between the effective connectivity values and FMA scores. Referring to the Automated Anatomical Labeling (AAL) atlas, the brain regions which were based on the location of the voxels showing significant differences (increased or decreased) in effective connectivity between each pair of the three groups were extracted as masks. The GCA value for each brain region was obtained by averaging the GCA value of each voxel within mask and correlated to the corresponding FMA score of the stroke patients, and the significant statistical threshold was set at P < 0.05.

## Results

### Effective connectivity from the ipsilesional M1 to the whole brain

**Fig 2:**
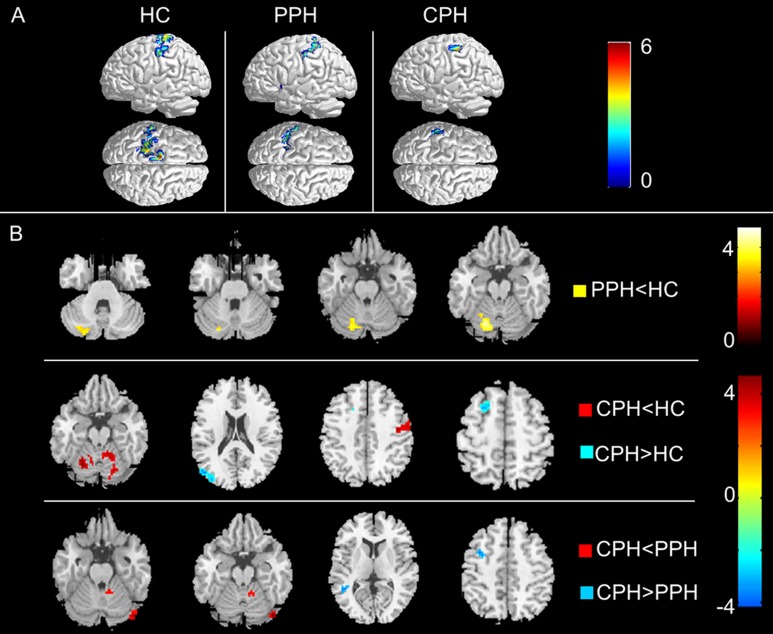
Effective connectivity from the ipsilesional M1 to the whole brain. (A) The within-group patterns of effective connectivity from the ipsilesional M1 to the whole brain. (B) The between-group differences in effective connectivity from the ipsilesional M1 to the whole brain. The statistical threshold was set at P < 0.01 with a cluster size > 1080 mm^3^ (P < 0.05, AlphaSim corrected). The color bar represents t-values. PPH: partially paralyzed hands; CPH: completely paralyzed hands; HC: healthy controls.

The within-group analysis showed that, according to the AAL atlas, the left M1 in the HCs had a causal influence on the ipsilateral sensorimotor cortices, including the primary sensorimotor cortex, supplementary motor areas (SMA), and ventral premotor cortex (PMv) (Fig 2A), which was consistent with the connectivity model for endogenous neural coupling reported in previous studies [16, 25]. However, from the visual inspection, in both PPH and CPH groups, the ipsilesional M1 (the left M1) lost its corresponding causal influences in this direction to a certain degree relative to the HC group. Specifically, the PPH group only showed effective connectivity from the ipsilesional M1 to the ipsilesional secondary sensory cortex, and the CPH group only showed effective connectivity from the ipsilesional M1 to a small portion of the ipsilesional secondary sensory cortex (Fig 2A).

For the between-group analysis, compared with the HCs, we found that the PPH group showed significantly decreased effective connectivity from the ipsilesional M1 to the ipsilesional cerebellum, while the CPH group exhibited decreases in effective connectivity from the ipsilesional M1 to the bilateral cerebellum and the contralesional PMv, and it showed increases in effective connectivity from the ipsilesional M1 to the ipsilesional superior frontal gyrus and occipital lobes. For the two subgroups, the CPH group showed the decreased effective connectivity from the ipsilesional M1 to the contralesional cerebellum, and the increased effective connectivity from the ipsilesional M1 to the ipsilesional middle temporal gyrus and middle frontal gyrus compared to the PPH group (Fig 2B and Table 2).

**Table 2:** Altered effective connectivity from the ipsilesional M1 to the whole brain.

Brain regions	BA	MNI Coordinates			Cluster size (mm^3^)	Maximum Z Score
		x	y	z		
**PPH<HC**						
Cerebellum_IL		-18	-69	-21	3726	4.84
**CPH<HC**						
Cerebellum_IL		-12	-60	-24	1485	4.41
PMv_CL	6	42	-12	48	1863	4.08
Cerebellum_CL		18	-90	9	3375	4.31
**CPH>HC**						
Occipital lobe_IL	19	-51	-75	24	2295	5.39
Frontal_Sup_IL	8	-18	-15	54	1323	4.96
**CPH<PPH**						
Cerebellum_CL		45	-81	-18	783	4.72
**CPH>PPH**						
Temporal_Mid_IL	39	-45	-51	6	540	4.10
Frontal_Mid_IL	6	-36	6	45	648	3.87

Abbreviations: PPH, partially paralyzed hands; CPH, completely paralyzed hands; HC, healthy controls; BA, Brodmann’s area; MNI, Montreal Neurological Institute; IL, ipsilesional; CL, contralesional; PMv, ventral premotor cortex; Temporal_Mid, middle temporal gyrus; Frontal_Mid, middle frontal Gyrus.

### Effective connectivity from the whole brain to the ipsilesional M1

**Fig 3:**
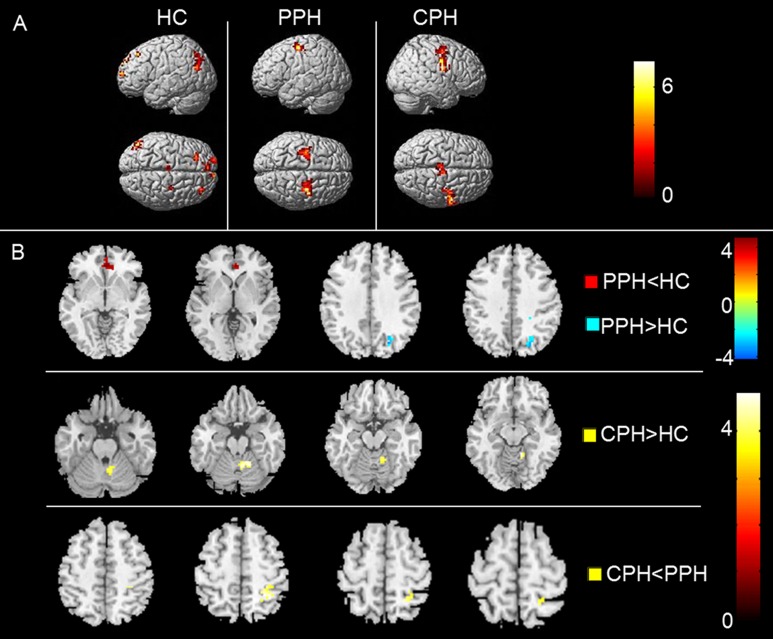
The effective connectivity from the whole brain to the ipsilesional M1. (A) The within-group patterns of effective connectivity from the whole brain to the ipsilesional M1. (B) The between-group differences in effective connectivity from the whole brain to the ipsilesional M1. The statistical threshold was set at P < 0.01 with a cluster size > 1080 mm^3^ (P < 0.05, AlphaSim corrected). The color bar represents t-values. PPH: partially paralyzed hands; CPH: completely paralyzed hands; HC: healthy controls.

The within-group analysis showed that, agreeing with previous findings [26], the left M1 in the HCs received influence from the fronto-parietal cortices, including the bilateral prefrontal cortex, left SMA, left posterior parietal cortex, and right PMv. We visually observed that, compared with HCs, the causal flow from the prefrontal cortex and the posterior parietal cortex disappeared in both the PPH and CPH groups. The PPH group showed effective connectivity from the bilateral dorsal premotor cortex (PMd) to the ipsilesional M1 and the CPH group showed effective connectivity from the bilateral SMA and contralesional PMv to the ipsilesional M1 (Fig 3A).

For the between-group analysis, compared to the HCs, the PPH group showed the decreased effective connectivity from the bilateral anterior cingulate gyrus (ACC) to the ipsilesional M1 and increased connectivity from the ipsilesional occipital lobe to the ipsilesional M1, while the CPH group only showed the increased effective connectivity from the contralesional cerebellum to the ipsilesional M1. Compared to the PPH group, the CPH group displayed decreased effective connectivity from the contralesional superior parietal lobe (SPL) to the ipsilesional M1 (Fig 3B and Table 3).

**Table 3:** Altered effective connectivity from the whole brain to the ipsilesional M1.

Brain regions	BA	MNI Coordinates			Cluster size(mm^3^)	Maximum Z Score
		x	y	z		
**PPH<HC**						
Anterior Cingulate_CL	32	3	39	-3	1107	3.92
**PPH>HC**						
Occipital lobe_CL	19	24	-72	45	1566	4.55
**CPH>HC**						
Cerebellum_CL		18	-51	-21	1350	4.37
**CPH<PPH**						
SPL_CL	40	27	-48	57	1080	3.97

Abbreviations: PPH = partially paralyzed hands; CPH = completely paralyzed hands; HC = healthy controls; BA = Brodmann’s area; MNI = Montreal Neurological Institute; IL = ipsilesional; CL = contralesional; SPL = superior parietal lobe.

### Correlation analysis between the altered effective connectivity and FMA scores

**Fig 4:**
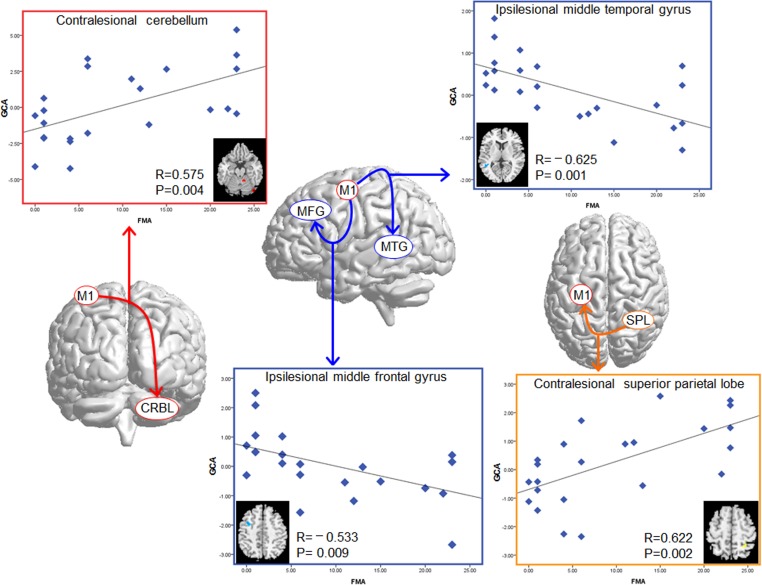
Correlation results between the GCA values of the altered effective connectivity in the CPH group compared with the PPH group, and the FMA scores of the stroke patients. The vertical axis indicates mean GCA values, and the horizontal axis indicates the FMA scores.

Significant correlations were revealed between the GCA values of the altered effective connectivity in the two subgroups of patients and FMA scores across all of the patients. The FMA scores were correlated with the GCA values of the contralesional cerebellum (R = 0.575, P = 0.004), the ipsilesional middle temporal gyrus (R = -0.625, P = 0.001), and the middle frontal gyrus (R = -0.533, P = 0.009), which showed differences in effective connectivity from the ipsilesional M1 between the CPH and PPH groups. In the altered effective connectivity from the whole brain to the ipsilesional M1 between the two subgroups of patients, the GCA values of the contralesional SPL (R = 0.622, P = 0.002) were positively correlated with the FMA scores (Fig 4). There were no correlations found between the the GCA values of the altered effective connectivity between patients group and HCs and FMA scores.

## Discussion

In the current study, we identified the abnormal causal flow of ipsilesional M1 in two subgroups of subcortical stroke. The causal information flow of the ipsilesional M1 was disrupted, mostly involving the motor-execution and fronto-parietal cortices, and this disruption was closely related to the severity of hand dysfunction. Subsequent correlation analyses revealed that the influence from the ipsilesional M1 to the contralesional cerebellum and from the contralesional superior parietal lobe to the ipsilesional M1 correlated positively with the FMA scores, whereas the influence from the ipsilesional M1 to the ipsilesional middle frontal gyrus and temporal gyrus corrected negatively with the FMA scores. These findings may provide new insights into the resting-state functional alterations of brain plasticity following stroke.

The present study revealed that the left M1 in the HCs had a causal influence on the ipsilateral sensorimotor cortices, which represent key motor areas underlying executing hand movements [27]. For feedback, the left M1 received influence from the fronto-parietal cortices, which are engaged in motor preparation and control [28–30]. Impressively, the causal flow from the prefrontal cortex and the posterior parietal cortex disappeared in both the PPH and CPH groups. These results are in accordance with the findings of Inman et al. [11] in stroke patients, which implied an altered resting-state effective connectivity of the fronto-parietal motor control systems on the primary motor network. Our findings add supporting evidence that the coordination of inputs from fronto-parietal motor control and outputs to motor-execution networks produces the motor behaviors that healthy individuals demonstrate in everyday life [31], and the disability of this coordination may significantly contribute to hand motor dysfunction in stroke survivors. It cannot be determined with certainty how a specific proportion of motor-execution and fronto-parietal motor control networks affects movement performance based on conventional task-based fMRI or some other non-directional resting-state functional connectivity analysis, as both of these networks are engaged during resting-state, motor imagery (MI), and motor execution (ME) [9,30,31]. By combining task-fMRI with GCA, recent studies in healthy subjects have revealed more information exchange during ME than during MI in the overlapped activated areas of both tasks, which were primarily distributed on the primary motor cortex and fronto-parietal cortex [32, 33]. Hence, the disappearance of the causal flow from the prefrontal cortex and the posterior parietal cortex to M1 in the patient group suggests that the hand dysfunctions in stroke patients may be more related to the disturbed MI networks. We further found that the SMA and premotor cortex (PMC) promoted influence on the ipsilesional M1, supporting the idea that the cortices of the second control center of movement, such as the SMA, and the PMC would compensate for the lost superior control of the distant fronto-parietal cortices [34–36]. In this study, by using voxel-wise GCA, the motor-execution network was found to operate independently in the direction from the left M1 to the other brain regions, and the executive-control network was separated from the opposite direction. Accordingly, it is easy to observe the alteration of the two networks in the PPH and CPH groups. Therefore, it is possible to provide an efficient way to evaluate the severity of damage in the motor-execution and fronto-parietal motor control network in stroke patient.

The cerebellum plays a vital role in motor recovery of stroke patients [37, 38]. Cerebellar damage produces disorders of fine movement, equilibrium, posture, and motor learning [39, 40]. In the present study, we found that the PPH group showed decreased connectivity from ipsilesional M1 to the ipsilesional cerebellum, and the CPH group displayed reduced connectivity from ipsilesional M1 to the bilateral cerebellum relative to the HCs. Moreover, the CPH group showed decreased causal flow to the contralesional cerebellum compared with the PPH group. These findings were in line with a previous task-fMRI study, which reported that stroke patients with good recovery have clear changes in the activation of the cerebellar hemisphere opposite the lesions, but patients with poor recovery do not show activation-related changes in the cerebellum over the time course of recovery [37]. Therefore, we speculate that the abnormal causal flow of the cerebellum may contribute to the motor recovery after stroke by improving the coordination of movement and motor learning [38]. Meanwhile, we found a significant positive correlation between FMA scores and the GCA value of the contralesional cerebellum from the causal flow of the ipsilesional M1, which indicates that the more severely the connectivity to the cerebellum is disrupted, the poorer the recovery of hand motor function is. Additionally, in contrast, the increased causal flow from the contralesional cerebellum in the CPH group compared with the HCs may be explained as a compensatory response to the decreased influence from the ipsilesional M1, but this compensatory mechanism needs to be confirmed in further studies.

In the between-group analysis, compared to the HCs, we also found a decreased influence from the ipsilesional M1 to the contralesional PMv in the CPH group, but not in the PPH group. This phenomenon suggests that the disrupted influence of the ipsilesional M1 is associated with the severity of the hand dysfunction. The functional interactions between the PMv and M1 are essential for hand motor function [41–43], although the PMv itself plays a key role in shaping the hand during grasping [44, 45]. Thus, we propose that the significantly decreased connectivity from the ipsilesional M1 to the contralesional PMv in the CPH group is likely related to the affected hand movement initiation [42], which could specifically contribute to the poor outcome of the hand function of the CPH group. In addition, the PPH group showed deceased effective connectivity from the ACC to the ipsilesional M1 compared to HCs. Whether the ACC plays a key role in motor recovery in stroke patients has not been determined; however, the abnormal connectivity from the ACC to the ipsilesional M1 may affect the motor-control function of the stroke patients by regulating the process from motor preparation to execution [46]. Moreover, a decreased influence from the contralesional SPL was observed in the CPH relative to the PPH group, and was positively correlated with the FMA scores across all stroke patients. The findings from previous studies suggest that the stronger connectivity between the ipsilesional M1 and contralesional SPL predicts a higher level of recovery at chronic stages of stroke [9, 11, 47]. Hence, the relationship between increases in contralesional SPL-M1 connectivity and motor improvements suggests a supportive role of parietal areas in motor recovery.

The increased influences from the ipsilesional M1 to the ipsilesional prefrontal gyrus and occipital lobe were found in the CPH group. These findings were consistent with a previous study that revealed the increased connectivity of the ipsilesional M1 with these ipsilesional brain regions [4] and increased activations in the prefrontal gyrus and occipital lobe were also demonstrated in some task-fMRI studies [36, 48]. This may reflect the abnormalities of motor network interactions after a stroke, as well as plastic changes that compensate for the impaired connectivity to the ipsilateral somatosensory cortex involved in movement execution. Additionally, we further found that the CPH group presented an increased causal influence on the ipsilesional middle temporal gyrus (MTG) located in the posterior temporal lobe. One previous fMRI study provided evidence that this area is characterized by the function of audio-motor processing [49]. The MTG is not regarded as a direct motor-related region, thus we speculate that this abnormal connection may also be a compensatory effect. Also, the significant negative correlations between the GCA value in both the ipsilesional middle frontal gyrus and ipsilesional MTG and the FMA scores across the patients further support our inference for the role of the two regions.

The current study has some limitations. First, the meaning of the GCA in resting-state effective connectivity is not fully understood, and it still has some shortcomings. The different hemodynamic delay is difficult to clearly capture when the sampling rate is as long as 2 s [50], and the slow dynamics of the BOLD signal at 2 s may lead to missing some of the rapid causal influences [51]. Whether it is best to low-pass filter the resting-state fMRI data for GCA is still unclear, and further studies are needed to clarify this meaningful point. However, many researchers believe that GCA indeed captures the time-directed influence between brain regions [20–23]. Further studies combining fMRI and electrophysiology are needed to clarify the association between effective connectivity and neuronal activity. Second, the sample size of each stroke subgroup is relatively small. Further studies of a larger population are needed to verify these findings. Finally, we only selected the ipsilesional M1 as the seed ROI. In future, we should select multiple brain areas in the motor network as ROIs to conduct a multivariate GCA to study the Granger causality of the motor network after stroke more extensively. And, the impact of smoothing over one signal should be considered in the GCA analysis.

## Conclusions

In conclusion, we characterized the abnormal directionality of influence both from and to the ipsilesional M1 in subgroups of stroke patients. Our results suggested that the altered effective connectivity of the ipsilesional M1 mainly involved brain areas participating in motor execution and advanced motor control, which were closely related to the severity of hand dysfunction. Future studies should evaluate how rehabilitative therapy changes the specific proportion deficits of the motor execution and frontoparietal motor control networks in interacting with M1, and this evaluation is an essential next step in learning how to improve the therapeutic intervention of stroke patients with hand dysfunction.

## Supporting Information

S1 Fig Lesion location of the 23 stroke patients enrolled in this study. (TIF)

S1 Table Illustration of Paralyzed Hand Function Assessment. The hand in dark denotes the affected hand.
(DOCX)
