# Supplementary figures and images for: Altered Effective Connectivity of the Primary Motor Cortex in Stroke: A Resting-State fMRI Study with Granger Causality Analysis
Source: PLoS One. 2016 Nov 15;11(11):e0166210. doi: 10.1371/journal.pone.0166210 (PMC5112988; doi:10.1371/journal.pone.0166210)

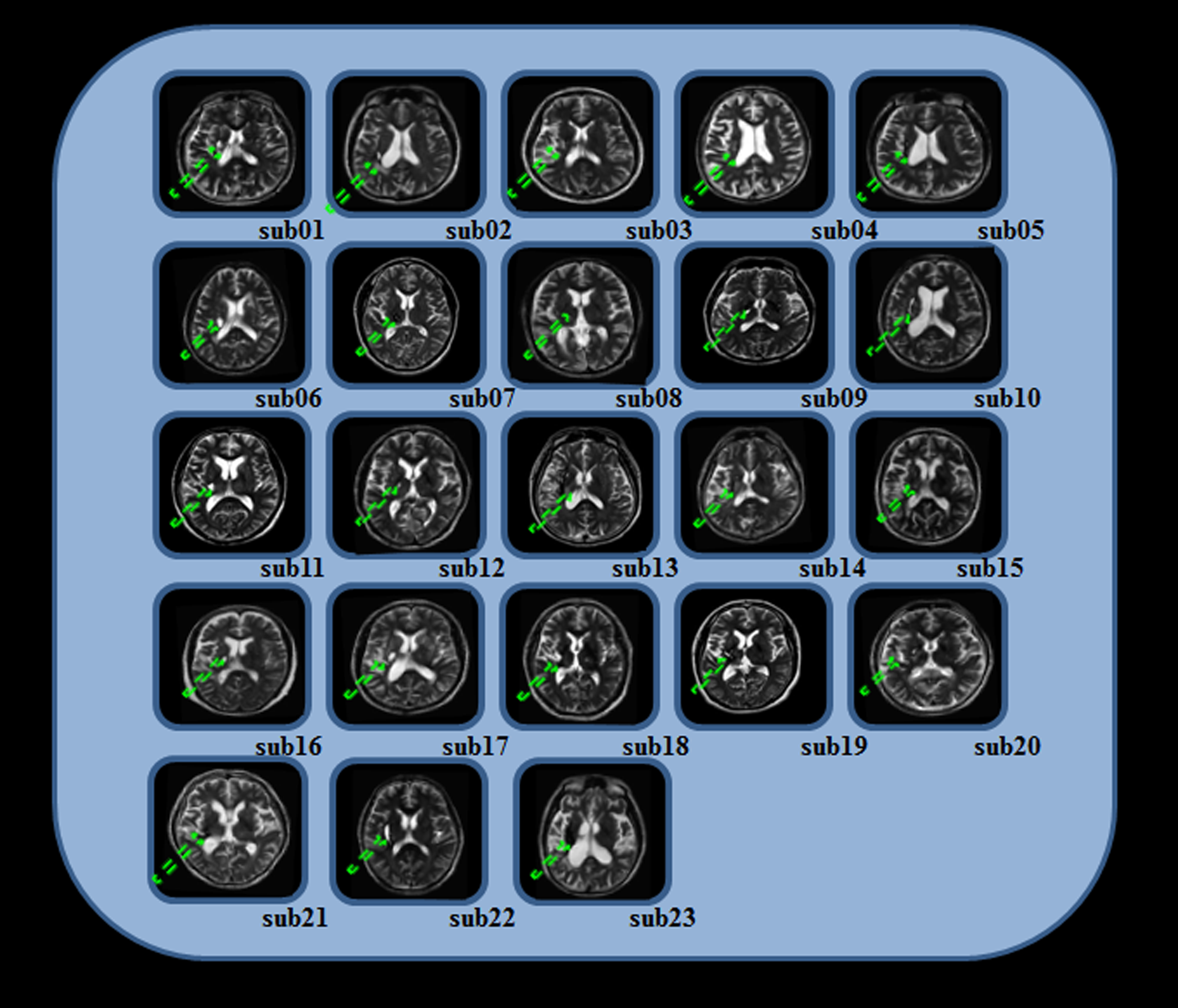

Supplement: S1 Fig — (TIF) [file pone.0166210.s001.tif]
