# Supplementary material for: Altered Effective Connectivity of the Primary Motor Cortex in Stroke: A Resting-State fMRI Study with Granger Causality Analysis
Source: PLoS One. 2016 Nov 15;11(11):e0166210. doi: 10.1371/journal.pone.0166210 (PMC5112988; doi:10.1371/journal.pone.0166210)
Supplement: S1 Table — The hand in dark denotes the affected hand. (DOCX) [file pone.0166210.s002.docx]

**S1 Table. Paralyzed Hand Function Assessment**

| **Action items** | **Illustration** | **Hand function classification** | **Evaluation criteria** |
| --- | --- | --- | --- |
| 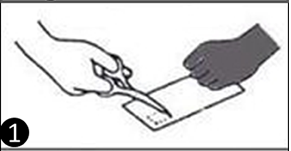 | The affected hand stabilizes a piece of paper on the table, with the unaffected hand controlling a shear to cut the paper | Crippled hand | Could not completed any activities |
| 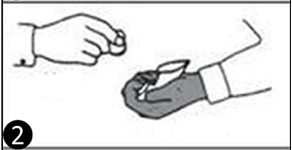 | The affected hand holds a wallet, with the unaffected hand taking a coin from the wallet | Assistant hand C | Finished one of five activities |
| 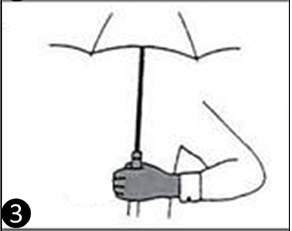 | The affected hand holds an unfolded umbrella in the air for at least 10 seconds | Assistant hand B | Finished two of five activities |
|  |  | Assistant hand A | Finished three of five activities |
| 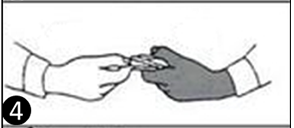 | The affected hand controls a nail scissor to trim nails of the unaffected hand | Practical hand B | Finished four of five activities |
| 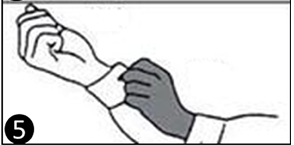 | The affected hand buttons the cuff of the unaffected side. | Practical hand A | Completed all of five activities |

**Note.** The hand in dark denotes the affected hand.
